# Structure and Growth Pattern of Pseudoteeth in *Pelagornis mauretanicus* (Aves, Odontopterygiformes, Pelagornithidae)

**DOI:** 10.1371/journal.pone.0080372

**Published:** 2013-11-14

**Authors:** Antoine Louchart, Jean-Yves Sire, Cécile Mourer-Chauviré, Denis Geraads, Laurent Viriot, Vivian de Buffrénil

**Affiliations:** 1 Centre National de la Recherche Scientifique, Unité Mixte de Recherche 5242, Institut de Génomique Fonctionnelle de Lyon, Equipe évo-dévo de la denture chez les vertébrés, Ecole Normale Supérieure de Lyon, Université Lyon 1, Lyon, France; 2 Université Pierre et Marie Curie, Unité Mixte de Recherche 7138 - Systématique, Adaptation, Evolution, Equipe évolution et développement du squelette, Paris, France; 3 Laboratoire de Géologie de Lyon, Terre, Planètes, Environnement, Unité Mixte de Recherche 5276, Centre National de la Recherche Scientifique, Ecole Normale Supérieure de Lyon, Université Lyon 1, Villeurbanne, France; 4 Centre National de la Recherche Scientifique, Unité Mixte de Recherche 7207 – Centre de Recherches sur la Paléobiodiversité et les Paléoenvironnements, Département Histoire de la Terre, Muséum National d'Histoire Naturelle, Paris, France; University of Birmingham, United Kingdom

## Abstract

The extinct Odontopterygiformes are the sole birds known to possess strong and sharp bony pseudoteeth, the shape and location of which are closely mimetic of real teeth. The structure of the pseudoteeth is investigated here in a late Pliocene/early Pleistocene species, *Pelagornis mauretanicus*, using X-ray microtomography and thin sections. The results are interpreted with regard to the pseudotooth mode of growth, and have implications concerning aspects of *Pelagornis* ecology. The larger pseudoteeth are hollow and approximately cone-shaped, and the smaller ones are rostro-caudally constricted. The walls of pseudoteeth are composed of bone tissue of the fibro-lamellar type, which is intensively remodeled by Haversian substitution. The jaw bones display the same structure as the pseudoteeth, but their vascular canals are oriented parallel to the long axis of the bones, whereas they are perpendicular to this direction in the pseudoteeth. There is no hiatus or evidence of a fusion between the pseudoteeth and the jaw bones. Two possible models for pseudotooth growth are derived from the histological data. The most plausible model is that pseudotooth growth began after the completion of jaw bone growth, as a simple local protraction of periosteal osteogenic activity. Pseudotooth development thus occurred relatively late during ontogeny. The highly vascularized structure and the relative abundance of parallel-fibered bone tissue in the pseudoteeth suggest poor mechanical capabilities. The pseudoteeth were most likely covered and protected by the hardened, keratinized rhamphotheca in the adult during life. The late development of the pseudoteeth would involve a similarly late and/or partial hardening of the rhamphotheca, as displayed by extant Anseriformes, Apterygiformes and some Charadriiformes. This would add support to the hypothesis of a close phylogenetic relationship between Odontopterygiformes and Anseriformes. The late maturation of the *Pelagornis* feeding apparatus, and hence the delayed capability for efficient prey catching, suggests that *Pelagornis* was altricial.

## Introduction

All living birds (Neornithes, approximately 9900 species) are toothless, and they represent 94% of all edentulous living tetrapods [1], [2]. This success corresponds to an unprecedented ecological diversification that was likely favored by edentulism itself and its correlates [2]. Thus, it is all the more surprising that a single, distinctive extinct avian clade, the Odontopterygiformes, developed bony pseudoteeth [3] resembling true teeth, a character most likely derived subsequent to neornithine edentulism [2]. This pseudodentition has peculiar characteristics, and displays an original distribution pattern with pseudoteeth of uneven sizes arranged in regular “waves” [3]–[9].

Odontopterygiformes lived above and around oceans and large seas almost worldwide from ca. 55 to 2.5 Ma [2]–[10]. They diversified into many species that have been placed in two generally recognized taxa: on the one hand most Neogene forms (placed in the family Pelagornithidae); on the other hand most Paleogene forms, either also included in the family Pelagornithidae or separated into a distinct family, the Odontopterygidae [8]–[10]. At least five genera are recognized, each comprising one to six species. The Odontopterygiformes are placed in the Neornithes, and are generally considered as Neognathae. They were previously considered to be close, or even to belong, to the Procellariiformes or to the Pelecaniformes (sensu [3], [11]), but this opinion was based on characteristics now viewed as convergent. The phylogenetic placement of the Odontopterygiformes remains unresolved, but seems to be basal, close to the Galloanserae (gamefowl and waterfowl) or even as the sister taxon to the Anseriformes (waterfowl) [12]–[14]. The occurrence of bony pseudoteeth, with diverse shapes of odontoids or only bumps (as in, for example, some amphibians [15]) is exceedingly rare among vertebrates. The only other bird clade displaying a series of bony odontoids, albeit much less developed and sharp than in the Ondontopterygiformes, were some of the recently extinct ‘goose-like’ moa-nalos of the Hawaiian Islands [16].

In addition to their unique pseudodentition, the mandibles of Odontopterygiformes show an intraramal hinge (streptognathism), the ventral two thirds of which consists of a synovial joint, and they also lack a bony symphysis [17]. The combination of these features has been proposed to have allowed the mandibular rami to bow considerably, and partly independently, in a horizontal plane, thus allowing large prey to be caught and ingested [17]. Such a feeding strategy seems to have been a key-factor in the evolution of the Odontopterygiformes, with a cascade of concomitant morphological and functional specializations bearing not only on their mandibular morphology, but also on their general skeletal structure [11], as well as on their size, locomotion specialization, and broad ecological adaptation [11], [13], [17]. Several Pelecaniformes show diverse degrees of development of an intraramal hinge; however, the precise type of hinge observed in pseudotoothed birds, as well as the lack of a bony symphysis, are otherwise known only in the Cretaceous toothed birds Hesperornithiformes and *Ichthyornis*. This similarity has been attributed to convergence [17]. A possible consequence of such a mandibular kinetism was to weaken the mandible and reduce its grasping strength. The acute pseudoteeth, as well as the premaxillary hook, are likely to have compensated for that lack of grasping strength in the Odontopterygiformes [17], as true teeth compensated for reduced grasping strength in the Cretaceous taxa. That hypothesis suggests that the pseudodentition was indeed a necessary adaptation to a particular conformation of the jaws, which is unknown in any other toothless neornithine bird, fossil or extant.

The histological structure of pseudoteeth has been investigated by previous authors based on two isolated pseudoteeth of *Pelagornis* (*Osteodontornis*) *orri*, a Miocene form from California [3], [18]. The pseudoteeth were interpreted as bony outgrowths of the jaw bone, devoid of mineralized dental tissue, and most probably covered by a rhamphotheca in life [3], [18]. The growth pattern and possible functional role of the pseudoteeth were not considered. In addition, some of the observations presented were ambiguous, unclear, or conflicting, such as those concerning the orientation of the vascular canals, or the presence of peripheral circumferential lamellae (primary bone tissue). These problematic issues remained unverifiable because it was not possible to relocate the original thin sections. Important questions thus persist regarding, for instance, the structure and growth pattern of the pseudoteeth, which have no equivalent today.

The present study, by providing a detailed histological description of pseudoteeth in *Pelagornis mauretanicus*, aims to: (i) propose a reconstruction of their growth pattern; (ii) assess their spatial relationships with the covering rhamphotheca, and the relative timing of pseudotooth growth and rhamphothecal hardening; and (iii) contribute to interpretations of some ecological traits of the Odontopterygiformes that might be influenced by pseudotooth growth.

## Materials and Methods

*Pelagornis mauretanicus* Mourer-Chauviré and Geraads, 2008, from the late Pliocene/early Pleistocene of coastal Morocco, is the geologically latest occurrence of a pseudotoothed bird known worldwide (*ca.* 2.5 Ma [7]; confidence interval estimated as approximately 2.7–2.3 Ma [DG] on the basis of by biochronological dating). A possibly contemporaneous Pacific record of a Pelagornithidae is dated with a confidence interval of 3.4–2.4 Ma (radiometric dating; [19]). With an estimated wingspan of 4–5 or perhaps 6 meters, *P. mauretanicus* was a very large pelagic bird like all members of the genus *Pelagornis* [7], [9], [11].

**Figure 1:**
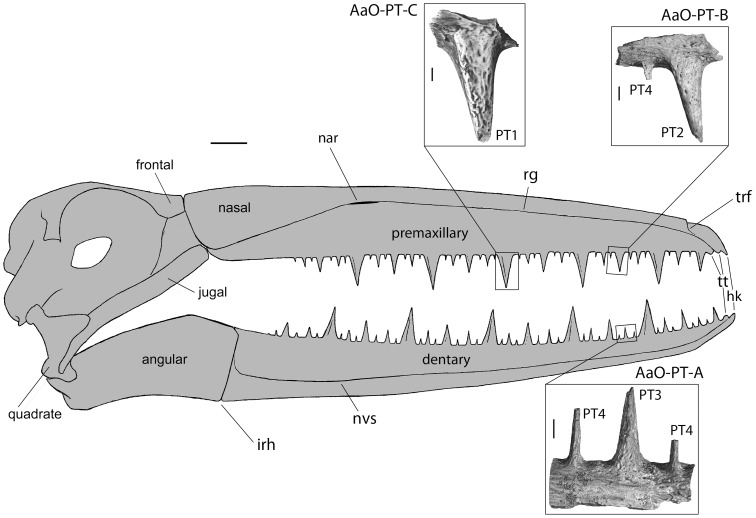
*Pelagornis mauretanicus* skull in right lateral view, showing the morphology and spatial organization of pseudoteeth. The line drawing of skull is a reconstruction using the general shape in species of *Pelagornis*. The fossils used in this study are shown magnified in the inserts, with indication of the ranking of the pseudoteeth, based on their relative size, shape and position. Given the fragmentary nature of our specimens, their precise location within or among jaw bones is not known, and their locations shown here are one example among other possibilities. It is however precisely shown to which part of a sequence the specimens belong, and their orientation (latero-medial and caudo-rostral), with the help of more complete series in [7]. hk, hook; irh, intra-ramal hinge; nar, outer narial opening; nvs, neurovascular sulcus; rg, rostral groove; trf, transverse furrow; tt, tomial “teeth” (from [3], [6], [7], [9], [17]). PT, pseudotooth. 1 to 4 indicate the rank of a pseudotooth. Main frame scale bar = 2 cm. Scale bar in inserts = 2 mm.

The paleontological material used in this study consists of three specimens of jaw bones of *P. mauretanicus* from Ahl al Oughlam, Casablanca, Morocco [7]. This paleontological locality is dated as late Pliocene/early Pleistocene (ca. 2.5 Ma). At the time of deposition, it consisted of a network of fissures and interconnected galleries in a jumble of calcarenite blocks at the foot of what was then a cliff on the shore [7]. The three fragments are recorded under the references AaO-PT-A, AaO-PT-B, AaO-PT-C, in the paleontological collections of INSAP (Institut National des Sciences de l'Archéologie et du Patrimoine) at Rabat, Morocco. Well-developed pseudoteeth occur on these bones (Fig. 1). Taphonomic information suggests that our three fossils are from a single individual. Indeed, among the various *Pelagornis mauretanicus* remains found at Ahl al Oughlam, only two elements are represented twice (distal part of right humerus; right pterygoid), and one element is represented three times (distal part of right radius). All other skeletal parts are represented only once, including all skull remains, which are thus likely to be parts of a single skull. Moreover, like the complete set of *Pelagornis* bone fragments that were associated at Ahl al Oughlam, the sizes, and non-fibrous surfaces of the skull bones are indicative of adult or subadult developmental stages [7]. In brief, we consider that the three jaw bone fragments studied here probably originate from a single fully (or nearly so) developed individual.

**Figure 2:**
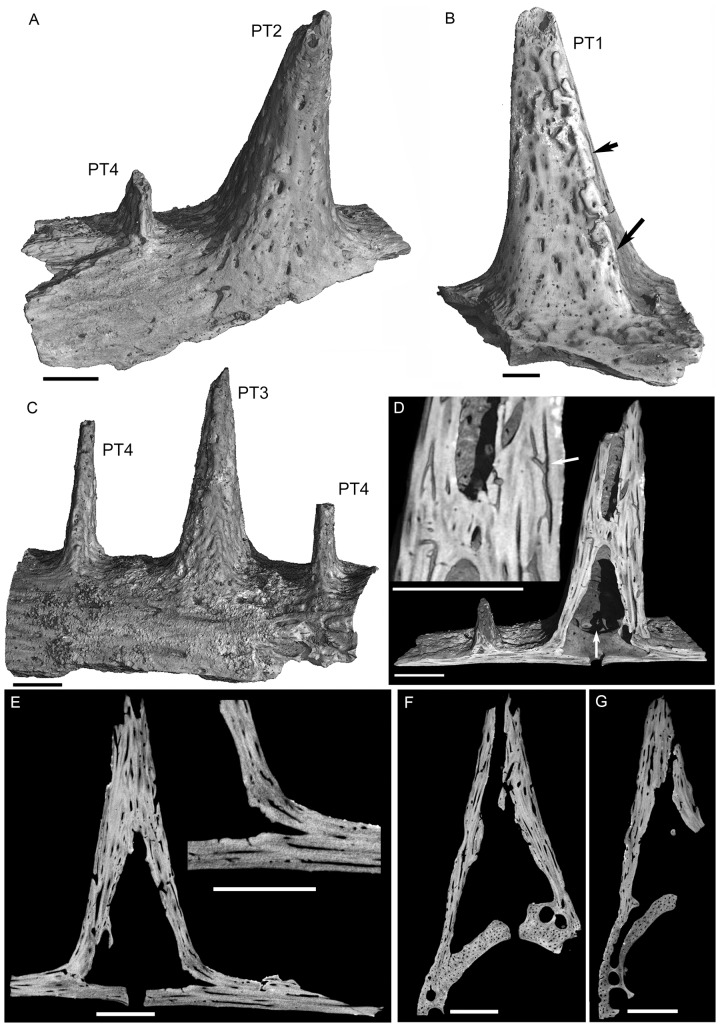
Microanatomical features of *Pelagornis* pseudoteeth as seen in computed tomographic reconstructions. (A) Specimen AaO-PT-B in caudo-medial view (and oblique from a slightly occlusal view), showing the morphology of second and fourth rank pseudoteeth (PT2, PT4) and the fragment of the jaw bone from which they developed. Note the density of vascular pits on the largest tooth, and the laterally shifted implantation of the rank 4 pseudotooth. (B) Specimen AaO-PT-C in lateral view, showing the well-marked caudo-lateral ridge (arrows) on the first rank pseudotooth. (C) Specimen AaO-PT-A in lateral view, showing the rank 3 and the two rank 4 pseudoteeth (one of them with more of the apex missing than the other). There is a small amount of sedimentary matrix still attached to parts of this specimen. (D) Virtual parasagittal section in AaO-PT-B showing the hollow inside of the pseudoteeth, and the large vascular pit perforating the basal plate (vertical arrow). The insert is an enlargement of the main view showing branching and anastomoses (arrow) in the vascular network. (E) Virtual parasagittal slice in specimen AaO-PT-B showing the hollow core of a large pseudotooth and the abundant vascular canals that run inside the basal plate, and from this plate occlusally to the pseudotooth walls. Note also the absence of any discontinuity between the pseudotooth and the jaw bone, with enlargement in the insert. The rank 4 pseudotooth of this specimen is visible tangentially on this slice because rank 4 pseudoteeth are positioned more laterally than the axis of larger pseudoteeth. (F) Virtual transverse slice in the rank 2 pseudotooth of specimen AaO-PT-B. (G) Virtual transverse slice in the rank 3 pseudotooth of specimen AaO-PT-A. Note in F and G the hollow core of the pseudotooth, the abundance of vascular canals in both the pseudotooth walls and the basal plate, and the difference in the orientation of the canals in the pseudotooth (vertical, dorso-ventral, i.e., basal-apical orientation) and in the basal plate (sagittal, rostro-caudal orientation). Scale bars = 2 mm.

The pseudoteeth were ranked from 1 to 4 depending on their size and position (Figs. 1, 2). Nomenclature of rank 1–3 pseudoteeth follows Mourer-Chauviré and Geraads [7] and corresponds to their “orders”, while rank 4 pseudoteeth correspond to the “needles” of these authors, and the “spines” of Howard [3]. One jaw bone fragment (AaO-PT-A) displays three pseudoteeth of different sizes. As compared with more complete series described in Mourer-Chauviré and Geraads [7], they appear to represent two pseudoteeth of rank 4 surrounding a pseudotooth of rank 3. Another fragment (AaO-PT-B) comprises two adjacent pseudoteeth: one of rank 2 and one of rank 4. A third specimen (AaO-PT-C) comprises a single pseudotooth, of rank 1 (Fig. 1).

These fossils were first imaged through conventional X-ray microtomography. These CT-scans were performed at General Electrics (GE) and at Ecole Normale Supérieure de Lyon with a GE Phoenix Nanotom 180 device, and analyzed using VG-Studio MAX 2.2 software. The following parameters were used for the different specimens (voltage, current, voxel size): 90 kV, 90 µA, 9.44 µm for AaO-PT-A; 90 kV, 100 µA, 10.56 µm for AaO-PT-B; 100 kV, 70 µA, 9.74 µm for AaO-PT-C; and 100 kV, 70 µA, 4.14 µm for a detail of AaO-PT-C.

For the virtual extraction of the vascular network of specimen AaO-PT-B, the vascular canals were selected through empirical thresholding followed by additional manual segmentation, using VG-Studio MAX 2.2.

For histological observations, sub-serial thin sections 80 to 100 µm thick were made from these samples at an interval of 1 mm, using the conventional techniques for this kind of preparation (see e.g. [20]). Three section planes were used: (1) a plane parallel to the long axis of the pseudotooth and to the longitudinal axis of the jaw bone, referred to here as the “sagittal” plane; (2) a plane parallel to the long axis of the pseudotooth but perpendicular to the longitudinal axis of the jaw bone, referred to here as the “transverse” plane; (3) a plane perpendicular to the long axis of the pseudotooth, referred to here as the “horizontal” plane. These sections were observed microscopically in ordinary and polarized transmitted light. The terminology used to describe bone tissues complies with the synthetic typology given by Francillon-Vieillot et al. [21].

Line drawings were made for each of the sections using a camera lucida with a precision of 40–50 µm, depending on magnification. These sketches were digitized using Photoshop CS v5, with bone tissue in black and cavities in white, in order to perform histomorphometric measurements using the software Image J. The main measurements were: (i) global bone compactness, which expresses the area occupied by mineralized bone tissue as a percent of total sectional area. This measurement can also be made in a selected part of a section; (ii) linear measurements bearing on various morphological details of the pseudoteeth, e.g. the thickness of their walls (i.e., cortices), or the width of their inner cavities, vascular canals, or superficial pits. Additional measurements were calculated using the CT volumes in VG-Studio MAX 2.2 (see Table S1).

We reserve the term pseudoteeth (also called bony teeth) for the well-developed structures of the Odontopterygiformes analyzed here; those observed in some moa-nalos, which are relatively much smaller and obtuse, should be called bony odontoids or bony serrations for distinctiveness.

Given the fragmentary nature of the jaw bone fragments that were attached to the pseudoteeth of our specimens, it was not possible to determine the regions of the lower or upper jaws to which they belong. Therefore, the fragments are merely referred to below as “jaw bone(s)”. Considering the regularity of the pseudodentition across both jaw tomia (i.e., occlusal margins of the jaws) (Fig. 1), this situation does not hamper the interpretation of our results.

The three series of thin sections are housed in the paleohistological collection of the Muséum national d'Histoire naturelle (MNHN - Histopal collection, in Paris, France), under the reference numbers MNHN-HISTOS 191 to 205. The CT data are deposited in AL's team (at IGFL, ENS de Lyon, France) and accessible for research on demand.

Ethics statement: No specific permits were required for the described study, which complied with all relevant regulations of the legislation of Morocco, concerning collection and study of fossils from this country (see [7] and all previous publications on fossils from Ahl al Oughlam). All information on fossils collected at this locality and permits can be found in the ca 70 articles co-authored by D. Geraads on fossils from this locality since the 1980–1990s.

## Results

### External morphology of pseudoteeth

The pseudoteeth of *P. mauretanicus* display different morphologies according to their rank order: those of ranks 1 to 3 are approximately sharp cones that reach up to ca. 20 mm in height and ca. 9 mm in basal diameter (rank 1; cf. Table S1). Rank 4 pseudoteeth resemble short blades and are located more laterally on the jaw bone tomia relative to the axes of higher rank pseudoteeth. The main axes of the pseudoteeth are inclined rostrally (relative to the jaw tomia) by some 5° to 15° for rank 1 pseudoteeth, less for ranks 2 and 3 pseudoteeth, and not at all for rank 4 pseudoteeth (our specimens and [7]). Consequently, the cone apices are off-centred in the same direction. Rank 1, and to a lesser extent rank 2, pseudoteeth tend to be slightly pyramidal, with a well-marked ridge situated caudo-laterally (Fig. 2A). Pseudoteeth of lower rank are progressively more “compressed” rostro-caudally. The bases of rank 1 pseudoteeth are slightly more elongated rostro-caudally than latero-medially. Conversely, the base of rank 2 pseudoteeth is slightly wider latero-medially than rostro-caudally, a tendency that is more accentuated in rank 3 pseudoteeth. Rank 4 pseudoteeth are at the end of the spectrum and display a blade-like shape, with a base that is almost twice as wide as long (Table S1).

The outer surface of pseudoteeth of all ranks, as well as that of jaw bones, is entirely perforated by vascular pits, the maximum diameters of which are up to 500 µm for the largest, elliptical ones (Fig. 2A–C). The spatial density of these pits is somewhat higher on the pseudoteeth themselves than on the surface of adjacent jaw bone (especially for the larger pits). Observed pit densities are, respectively: 1.02 pits/mm^2^ vs. 0.93 for the pits from 100 to 200 µm in diameter; 0.73 pits/mm^2^ vs. 0.46 for the pits >200 µm diameter.

Although all pseudoteeth examined displayed a broken apex, this is due to post-depositional breakage (absence of healing or bone regrowth). The outer surfaces of the pseudoteeth are void of cracks or chipping traces (e.g. Fig. 2A–C), and instead display the fine relief due to vascular pits. Whatever the size or shape of the pseudoteeth, they are in complete continuity with the supporting jaw bone, and there is no hiatus, groove nor any kind of visible relief that could have resulted from their joining to the jaw bone following their formation.

### Inner micro-anatomy of pseudoteeth

A pseudotooth - the empty pseudo-cone, or blade, depending on rank - is closed by a horizontal osseous floor (named here “basal plate”). The basal plate of large pseudoteeth is pierced by a large foramen (average diameter ca. 0.45 mm in one rank 2 pseudotooth; ca. 1.7 mm in one rank 1 pseudotooth; Table S1). This basal foramen allows communication between the hollow interior of a pseudotooth and the hollow medullary region of the jaw bone (Fig. 2D–F). The smaller, rank 3 and 4 pseudoteeth bear no such large foramen in their basal plate.

**Figure 3:**
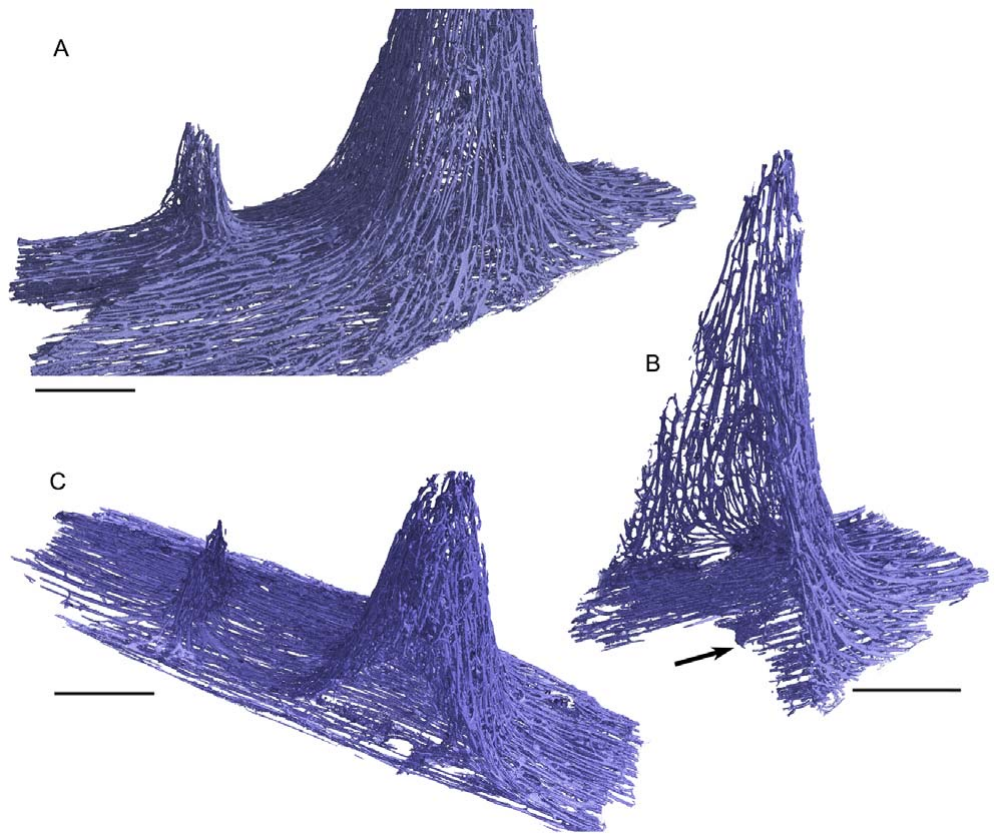
Virtual reconstruction of the vascular network in specimen AaO-PT-B showing ranks 2 and 4 pseudoteeth. A few of the widest and of the thinnest canals do not appear here due to the used parameters of extraction of the vascular canals network, but this does not modify the general view. (A) Caudo-medial detail view. (B) Transverse section in the rank 2 pseudotooth. (C) Parasagittal section in more oblique, partly occlusal and medial view. The dense network of canals extends continuously from the basal plate occlusally to the walls of the pseudoteeth. The canals of the basal plate are clearly sagittal (arrow in B) as the other canals of the jaw bone. Some of them are clearly inflected occlusally to colonize the walls of the pseudotooth. Scale bars = 2 mm.

**Figure 4:**
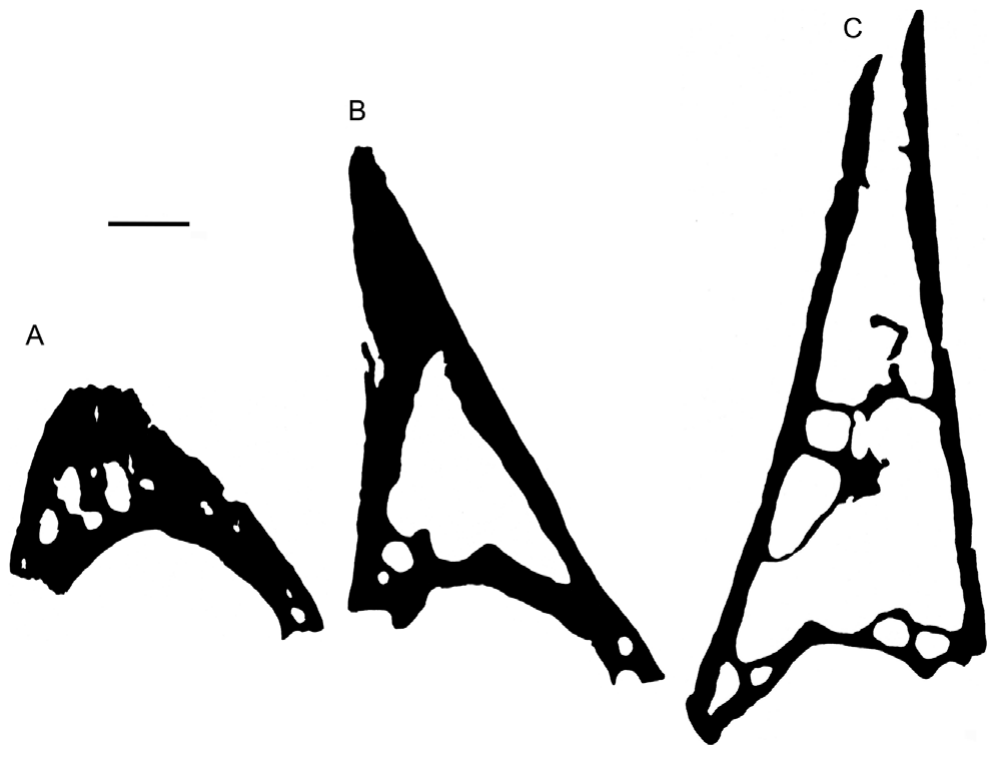
General architecture of different pseudoteeth, as seen on transverse thin sections. (A) Pseudotooth of rank 4 in specimen AaO-PT-A. (B) Pseudotooth of rank 3 of specimen AaO-PT-A. (C) Pseudotooth of rank 1 in specimen AaO-PT-C. Wall thickness is proportionally thinner, and hence total pseudotooth compactness lower, in larger pseudoteeth. Scale bar = 2 mm.

As revealed by the thin sections and microtomographic images, the greater part of the internal volume of the pseudoteeth consists of cavities (Figs. 2D–G, 3, 4). In the large, conical pseudoteeth there is a single cavity representing up to 60% of the total area (excluding the basal plate) in sagittal sections (Fig. 2D,E). Small (rank 4) pseudoteeth display several lacunae forming less than 20% of the total area in sagittal sections (Fig. 2D,E, 4C). The global compactness of the pseudoteeth (basal plate excluded) ranges from ca. 36% (rank 1 pseudotooth) to 80% (rank 4 pseudotooth) (Fig. 4). Pseudotooth cortices are greatly variable in thickness. They occupy most of the volume in rank 4 pseudoteeth, whereas they are proportionately much thinner in larger pseudoteeth (Fig. 4, Table S1). Mean cortex thickness represents some 20% to 30% of the basal pseudotooth radius in large pseudoteeth. Resorption lacunae are few inside the cortices, but bone vascularization is abundant, with a mean density of vascular canals of ca. 22–26 canals/mm^2^ (rank 1 pseudotooth). In the basal plate (same pseudotooth) there are ca. 25 canals/mm^2^. The mean diameter of vascular canals, from 20 µm (deep ones) to 45 µm (superficial ones), is similar in pseudotooth cortices and in basal plates. In the rank 1 pseudotooth the inner cortical compactness is 88% to 92%. The rank 3 pseudotooth (medium sized) displays an inner, cortical compactness of 89–90%, to be compared with a greater inner, cortical compactness (95–97%) for its basal plate and adjacent jaw bone (Table S1). This difference is due to a slightly higher vascularization in the pseudotooth itself (number and size of canals) compared with that of the jaw bone (basal plate included). This is in agreement with the slightly higher surface pitting observed on the surfaces of the pseudotooth versus jaw bone.

Pseudoteeth and basal plates clearly differ in the orientation of the canals. In the basal plate they are aligned in a sagittal direction as in the rest of the jaw bone, whereas they are oriented in a roughly perpendicular direction (pseudotooth basal-apical direction) in the pseudotooth cortices (Figs. 2D–G, 3). The tridimensional reconstruction of the vascular network in the basal plate and in the pseudotooth cortices show frequent anastomoses and transverse links between vascular canals, as well as the emergence of the canals at the external surfaces of the pseudoteeth (Fig. 3).

### Histological features of pseudoteeth

**Figure 5:**
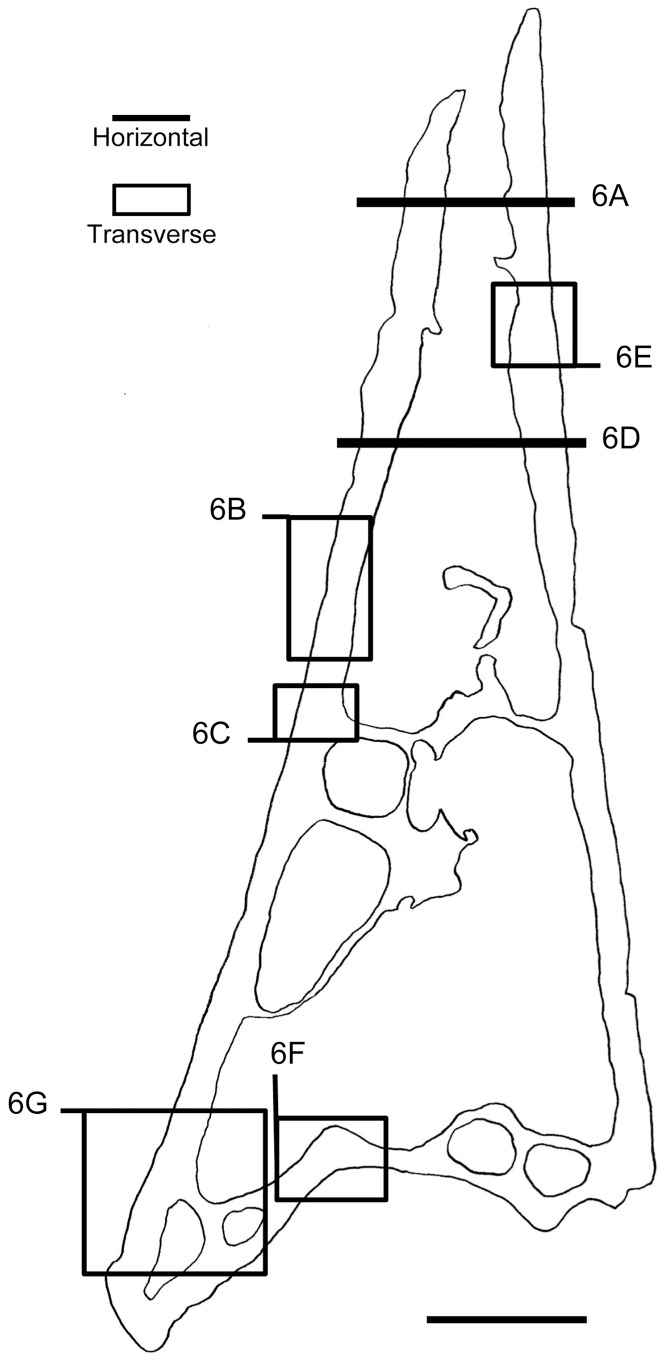
Location and orientation of the sections shown in Figure 6. Thin sections (A–C, E–G) and one virtual slice (D) were made in the rank 1 pseudotooth of specimen AaO-PT-C. The letters are those used in Figure 6. Scale bar = 2 mm.

**Figure 6:**
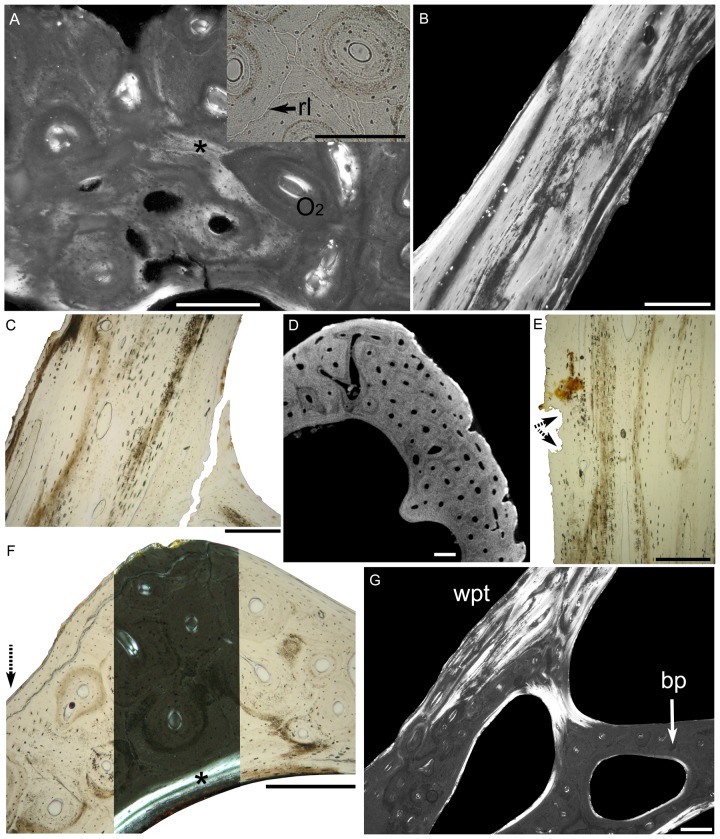
Histological features of the rank 1 pseudotooth of specimen AaO-PT-C. (A) Horizontal thin section in the apical region. Main frame: polarized transmitted light; insert: ordinary transmitted light. Remnants of primary bone tissue (asterisk) are scarce, whereas longitudinal primary and secondary (O_2_) osteons are abundant and appear monorefringent. Reversion lines (rl) are clearly visible around the secondary osteons. (B) Transverse thin section in the walls of the pseudotooth. Polarized transmitted light. The osteons are brightly birefringent, which reflects the longitudinal orientation of their collagen fibers. (C) Transverse thin section in the pseudoteeth walls. Ordinary transmitted light. Osteocyte lacunae have a spindle-like morphology in the parallel-fibered bone forming the osteons. There is an artefactual wrenching at the lower right of the image. (D) Virtual horizontal slice (microtomographic) showing differences in the mineralization rate of the osteons. (E) Transverse thin section in ordinary transmitted light. Howship's lacunae (arrows) on the deep side of the wall. (F) Aspect of the basal plate viewed in a transverse thin section. Polarized (centre of the section) and ordinary (lateral parts) transmitted light. Osteons (mostly secondary) are oriented sagittally. Some superficial resorption occurred locally (arrow), whereas the deep face of the basal plate was partly reconstructed by endosteal deposits (asterisk) after resorption. (G) Longitudinal thin section. Polarized transmitted light. The osteon orientation creates a sharp distinction between the basal plate (bp) and the walls of the pseudotooth (wpt). Scale bars = 200 µm.

There are no dental tissues (dentine, enamel, cement, periodontal tissues) in the pseudoteeth we examined. Figure 5 shows the location and orientation of the sections described below and presented in Figure 6. Pseudoteeth cortices are entirely made of bone tissue in the form of a fibro-lamellar complex (defined in [21]), which shows evidence of intense remodeling by Haversian substitution. The primary periosteal tissue is represented by discrete remnants, located between primary and secondary osteons, and more abundant in the apical region of the pseudoteeth than toward their base. In polarized light, this tissue shows a slight, irregular mass birefringence (Fig. 6A). It contains randomly distributed globular osteocyte lacunae with poorly developed canaliculi. These characteristics are intermediate between parallel-fibered (mass birefringence) and woven-fibered (shape and position of the osteocyte lacunae) osseous tissues. This condition is atypical for fibro-lamellar complexes, in which the primary periosteal bone is generally of well-characterized woven-fibered type. There are no visible anchoring or Sharpey's fibers. The primary and secondary osteons are parallel to each other and to the pseudotooth surface. Their walls are not made of true lamellar bone tissue as is the most common condition in vertebrates, but of parallel-fibered tissue that appears monorefringent in horizontal sections (Fig. 6A), and strongly birefringent in sagittal and transverse sections (Fig. 6B,G). This aspect in polarized light is due to a strong anisotropy of the once present collagen network, the fibers of which (and now the hydroxyapatite crystals) were all oriented parallel to the sagittal axis of the osteons. In the osteon walls, osteocyte lacunae are spindle-like and oriented in the same direction as were the collagen fibers before fossilization (Fig. 6C,E). This type of lacunae is characteristic of parallel-fibered tissue. In the virtual sections sampled from 3D reconstructions, neighboring secondary osteons can show different mineralization rates (Fig. 6D). This indicates that they formed at different times (the least mineralized ones being ontogenetically the most recent) and that the process of Haversian remodeling occurred progressively throughout the bird's life. However, the scarcity of erosion bays suggests that Haversian remodeling had ceased or had strongly slowed down at the growth stage reached by our specimen.

The surface of the pseudotooth walls facing the inner cavities displays, by places, an irregular, finely indented contour, which is characteristic of Howship lacunae (Fig. 6E). The latter were created by osteoclastic activity during phases of bone resorption. In the apical region of the pseudoteeth, Howship lacunae are essentially localized on the apical, and to a lesser extent, lateral walls of the internal cavities. In contrast, the basal walls of these cavities show signs of partial reconstruction, as illustrated by the occurrence of thin layers of endosteal lamellar bone. The situation is reversed in the basal region of the pseudoteeth, where resorption is directed towards the basis of the lacunae (Fig. 6F), whereas reconstruction occurs on apical and lateral walls (Fig. 6G). These observations show that the internal cavities of the pseudoteeth resulted from a process of bone resorption, and that the latter accompanied the growth of the pseudoteeth in apical and lateral directions.

The basal plates of pseudoteeth display the same basic histological structure as the pseudoteeth themselves. However, the secondary osteons are contiguous and no remnants of primary bone tissue can be identified (Fig. 6F), as is also the case in adjacent jaw bone parts. As mentioned above, these osteons are orthogonal (caudo-rostral direction) to those in the pseudoteeth (dorso-ventral direction). In cross sections of jaw bone, osteons appear round and monorefringent in polarized light, whereas the osteons in the pseudoteeth are birefringent and slender (Fig. 6F,G).

Histological observations confirm the absence of any discontinuity, fused or not, between the jaw bone tissue and the pseudotooth tissue. The vascular canals and the osteons of the jaw bone extend without interruption in the pseudoteeth, the only modification being a steep change in their orientation as mentioned above (Figs. 2D–G, 3). Therefore, pseudoteeth must be considered as outgrowths, or localized excrescences, of jaw bones.

The inner (deep) cortical surface surrounding the central cavity of the jaw bone is covered with a thin and irregular layer of endosteal bone (Fig. 6F). In transverse sections a reversion line separates this layer from the underlying jaw bone. This indicates that the jaw bone had been submitted to an extensive process of internal resorption progressing upward (lower jaw) and downward (upper jaw) towards occlusal surfaces (and probably also in all other directions) during growth, before being partly reconstructed by endosteal deposits.

## Discussion

### Comparison with previous studies

In terms of most of their histological characteristics, the pseudoteeth of *Pelagornis mauretanicus* are similar to those of *Pelagornis orri* described, albeit in much less detail, by Howard [3] and Howard and White [18]. In both species, the pseudoteeth are made of a bone tissue intensely remodeled by the Haversian process, and housing numerous vascular canals that open at the cortical surface. However, the interpretive sketch of a thin section provided in Howard ([3]: fig. 5), shows “circumferential lamellae” that are observed neither on the jaw bones nor on the pseudoteeth of our *P. mauretanicus* specimens. These lamellae obviously correspond to the external circumferential lamellae (also called external fundamental system), typically present in fast growing organisms (mammals, birds, etc.) at the periphery of the bones that have completed their growth in diameter or thickness. The intense Haversian remodeling displayed by the bone samples studied here confirms that they were all from subadults or adults; therefore, the lack of external circumferential lamellae might suggest that their ontogenetic age was somewhat less advanced than that of the *P. orri* specimen examined by Howard [3]. In their study of additional material of *P. orri*, Howard and White [18] elaborated on the presence of circumferential lamellae, but the thin section photograph that they provided ([18]: fig. 4) is inconclusive, being too dark and too low resolution to assess. It could nevertheless be possible, in theory, that the pseudoteeth display different growth dynamics in these two species (e.g., in relation with their size or their position on the jaw bone); however, there is no unambiguous evidence supporting this hypothesis.

Another difference between our observations in *P. mauretanicus* and those by Howard [3] in *P. orri* could relate to the orientation of the secondary osteons. In *P. mauretanicus* primary or secondary osteons are parallel or sub-parallel to the long axis of the pseudoteeth. In figure 5 of Howard [3], the orientation of the section is not indicated but, given the geometry of the jaw bone outlines, the sectional plane is obviously parasagittal. However, in this interpretive figure, most secondary osteons are drawn orthogonal to that plane, and to the long axis of the pseudoteeth. If real, such an orientation would be in conflict not only with our observations on *P. mauretanicus*, but also with all data available on the orientation of secondary osteons in bones (e.g., [22]).

For these reasons (i.e., the problems of vascular orientation, and of circumferential lamellae), the “reconstruction” (sic) proposed by Howard [3], a drawing of thin section that is the only illustration provided, must be considered with caution, and appears to be inaccurate. The thin section illustrated by Howard and White [18], although at a low resolution and dark, appears more in agreement with our observations on vascular canal orientation. Unfortunately, the original series of thin sections made of *P. orri* specimens by these previous authors [3], [18] is presently lost. Hence, the questions and doubts raised here, concerning the vascular orientation, as well as the circumferential lamellae, will remain unanswered, at least until *P. orri* is re-sampled.

### A model for pseudotooth growth

In the absence of pseudoteeth in living birds, and of fossils forming a growth series in *P. mauretanicus*, the growth pattern of the pseudoteeth can be inferred only from the histological details displayed by the bones of adult specimens. Two main characteristics must be considered as a base for all interpretation attempts: (1) the relationships between pseudoteeth and adjacent jaw bones; and (2) the opposite processes of bone accretion and resorption occurring at the surface of and/or inside the pseudoteeth. These characteristics create conceptual prerequisites that constrain the reconstruction of the pseudotooth growth pattern.

As in the bones of most ornithurine birds (e.g., [23]–[25]), pseudoteeth and adjacent jaw bone are composed of a heavily remodeled fibro-lamellar complex. Pseudoteeth are neither fused nor secondarily attached to the adjacent jaw bone (unlike, for instance, acrodont teeth), but are part of it. Therefore they are outgrowths of the jaw bone surface. This observation implies that, at the level of each pseudotooth, the only surface by which the jaw bone grows is the surface of the pseudotooth itself. The development of a pseudotooth is thus a local aspect of the growth in thickness of the jaw bone cortex; conversely, the growth in length or the sutural expansion of jaw bones are not (or marginally) involved in pseudotooth differentiation and growth. This is the first, most basic, constraint on any reconstruction of pseudotooth growth. This constraint has an important, practical consequence: modeling must primarily be considered in the transverse sectional plane that best reveals the modalities and circumstances of cortical thickening.

Transverse sections of pseudoteeth and adjacent jaw bone show two distinct and independent erosion fronts: one which created the broad cavity in the core of jaw bones during growth; the other which made the core of the pseudoteeth hollow. Since a blade of bone (the basal plate) separates these two erosion fronts, they could not have developed synchronously, even if the second front was much less active than the first one, because in this situation, the basal plate, resorbed from below and from above, would have disappeared in the earliest stages of growth. Therefore, these data indicate that the erosion front inside a pseudotooth appeared late in ontogeny, when the resorption front inside the adjacent jaw bone had nearly stopped. This is the second constraint on growth pattern reconstruction.

**Figure 7:**
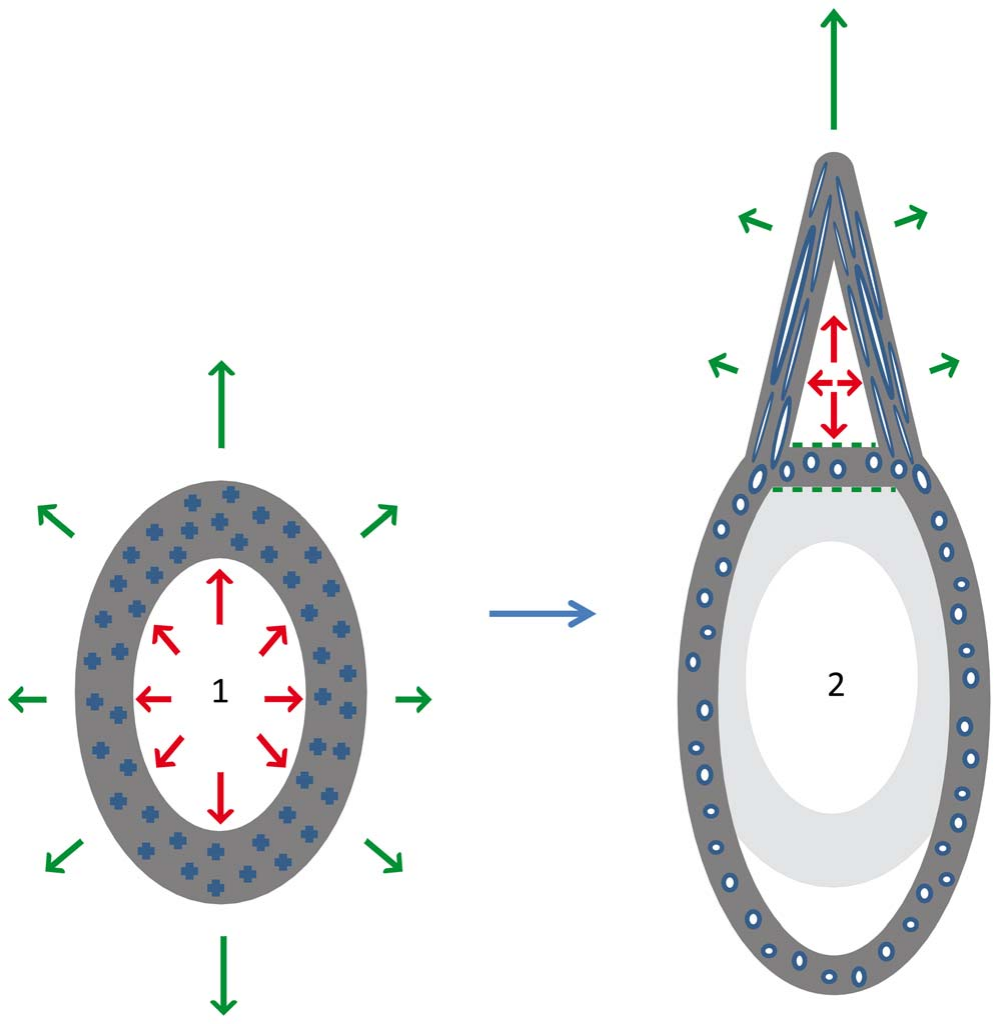
Schematic reconstruction of the first hypothesis for pseudotooth growth. The basal plate is totally part of the jaw bone, and pseudotooth growth occurs after completion of jaw bone growth. Two growth stages are represented: (1) early stage, before pseudotooth growth; (2) late stage, when the pseudotooth is growing. There would be no acceleration, but a simple protraction of bone accretion to form the pseudotooth. In stage 2 the light grey areas are the ancient, now resorbed, states of the bone at stage 1. Green arrows show the directions of bone accretion and red arrows the directions of bone resorption; both are longer when the phenomenon is of greater amplitude. The crosses within the jaw bone indicate that the orientation of primary vascular canals at growth stage 1 is unknown. In the pseudotooth (growth stage 2), the elongated oval segments, blue with white centre, represent the basal-apical (occlusal) orientation of primary and secondary osteons. In transverse section, they are cut along their elongation axis. The small blue circles with white centres in the jaw bone (basal plate included) represent the rostro-caudal orientation of secondary osteons at growth stage 2. In transverse section, they are cut orthogonally to their elongation axis. Green dashed lines show areas of secondary (reconstructive) endosteal bone deposits.

Two alternative models for the growth of a pseudotooth and adjacent jaw bone can now be considered. The first one is based on the postulation that the basal plate is not a part of the pseudotooth, but belongs to the jaw bone, because the basal plate and adjacent jaw bone are exactly identical in all their structural characteristics, and in perfect anatomical continuity. The growth model resulting from this postulation is that the pseudotooth developed as an excrescence on the jaw bone surface, after the latter had ceased, or completed most of, its own growth. This model can be developed as follows (Fig. 7). At early stages of post-hatching development, the occlusal (tomial) surface of the jaw bone presented no excrescence that could be indicative of pseudotooth initiation. By the end of jaw bone growth, the pseudotooth began its development due to a simple protraction in time, at a local scale, of the osteogenic activity of periosteal osteoblasts (Fig. 7-2), while this activity had ceased in other jaw bone territories. There is no need to assume that the pseudotooth resulted from an increase in sub-periosteal accretion speed, a hypothesis that would not be consistent with our histological observations. Indeed, the primary periosteal tissue observed in the pseudoteeth apex, a type of tissue intermediate between parallel-fibered and woven-fibered bone tissues, attests to a moderate growth speed (likely between 10 and 30 µm/day, according to experimental data in *Anas platyrhynchos* [23]), at least for the late growth stages of the pseudoteeth. While the pseudotooth developed, an extensive inner resorption field resulted in the hollowing of its core. Precursors of the osteoclasts involved in this process could have been brought *in situ* by capillary blood vessels penetrating the pseudoteeth through both the large foramen in the center of the basal plate and the numerous foramina located at the external surface. This resorption process ended when the inner geometry of the pseudotooth was completed. It is noteworthy that, for this model, the orientation of secondary osteons in the jaw bone, including the basal plate, is not an important characteristic: it could have been identical to that (unknown in our specimens) of the primary osteons that were in place before Haversian systems, or very different. A drastic change in vascular canal orientation when secondary osteons replace primary ones occurs frequently, as exemplified by, e.g., the replacement of radiating primary osteons (a juvenile feature) by longitudinal secondary osteons (an adult feature) in the long bones of King Penguins, *Aptenodytes patagonicus* [26]–[28]. Such a change in the geometry of the vascular network of the jaw bone, basal plate included, would be of no incidence on the growth model presented above.

**Figure 8:**
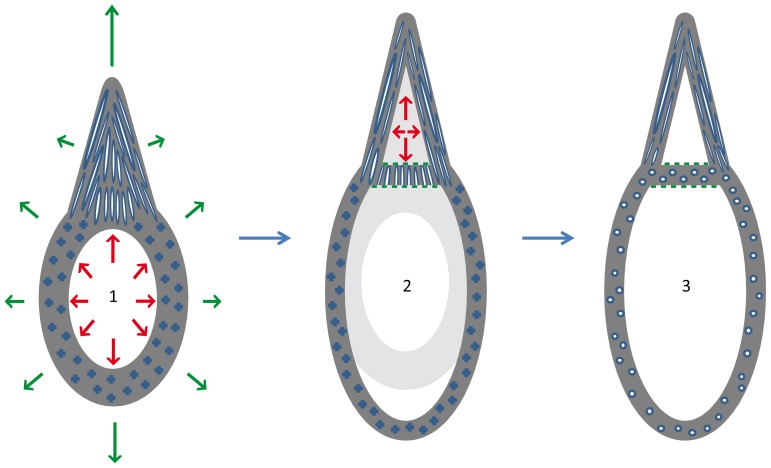
Schematic reconstruction of the second hypothesis for pseudotooth growth. The basal plate is part of the pseudotooth, and the latter grows simultaneously with the jaw bone. In an early growth stage (1), the pseudotooth and the jaw bone are actively growing, but sub-periosteal accretion is faster on the pseudotooth, which results in its differentiation from the subjacent jaw bone. At this stage, the pseudotooth is a solid cone with primary osteons sub-parallel to the main growth direction. When local growth is ending (2), a resorption field inside the pseudotooth creates a broad cavity, and provokes the differentiation of the basal plate. At this stage, the vascular canals within the basal plate still have their original orientation. In a late growth stage (3), extensive remodeling in the jaw bone and in the basal plate creates longitudinally oriented secondary osteons. Same symbols and color code as for Figure 7.

The second model (Fig. 8) postulates that the basal plate is a part of the pseudotooth. If so, the pseudotooth could have developed synchronously with the adjacent jaw bone, and would have merely represented, at a very local scale, its occlusal surface since the beginning of growth (Fig. 8-1). Considering the second constraint quoted above, it must be supposed that, in this situation, the pseudotooth was first a solid cone devoid of large inner cavity. In late growth stages, when the jaw bone had approximately reached its final size (at least in a transverse plane), the differentiation of the basal plate occurred as a result of the onset and spreading of the broad erosion field located in the core of the pseudotooth (Fig. 8-2). This model necessarily implies that the orientation of the vascular canals (most likely primary osteons) originally present in the basal plate was changed during the remodeling process of the plate (Fig. 8-3). Indeed, as described above, the vascular canals housed in pseudotooth walls are both primary and secondary osteons, parallel to each other and to the main growth direction of the pseudotooth (basal-apical, i.e., occlusal). It is therefore most likely that all the vascular canals that the pseudotooth housed during the various stages of its growth had the same general orientation as represented on figure 8-1. For unknown reasons, Haversian remodeling would have subsequently changed this orientation in the basal plate only. This model has another implication: the differentiation of a pseudotooth from the adjacent jaw bone during growth could only result from an important, local acceleration in sub-periosteal accretion. However, our histological observations provide no evidence of such acceleration.

Both growth models described above are possible, and only the histological study of different growth stages in Odontopterygiformes (at least a juvenile and an adult) could allow the question to be deciphered with certainty. However, it is noteworthy that the second model is much less satisfying because it involves three additional, unverified or problematic hypotheses that are unnecessary for the first model. The first hypothesis is a late onset of the resorption front inside the pseudotooth. Of course, this hypothesis has no necessity in the first model because the differentiation of the pseudotooth itself is late. The second hypothesis is that Haversian remodeling creates a total inversion in the direction of vascular canals in the basal plate. Although not impossible, this hypothesis would raise a number of unanswered (and presently unanswerable) questions. The third hypothesis is that the growth of a pseudotooth would result from accelerated local accretion. This hypothesis is not substantiated by histological data.

An additional comparative element can shed some light on the chronology of pseudotooth development. The only known jaw of a juvenile pseudotoothed bird is from an Eocene species, *Lutetodontopteryx tethyensis* [29]. In this specimen, the larger pseudoteeth (ranks 1 and 2) are well grown and the jaw bone has reached the same development in height (in the transverse plane) as that of an adult of the same species (comparison possible with position of the “neurovascular furrow”; [29]: fig. 2). However, the rank 3 pseudoteeth are not yet grown, which is evidence that these pseudoteeth, at least, did not develop synchronously with the jaw bone, but subsequently. This situation is more in favor of our first than of our second growth model, even though similar evidence is still lacking regarding rank 1 and 2 pseudoteeth, for which jaw specimens representing an earlier juvenile stage would be needed. Apparently, the growth in length of the jaw bone of the existing juvenile specimen of *L. tethyensis* was not fully complete when it died (again in comparison with the adult specimen assigned to the same species). There is no contradiction between this observation and our first growth model because the growth in length of bones (sutural or endochondral growth), is a process quite distinct from their growth in diameter or thickness (periosteal accretion), and pseudotooth development is related only to the second process, as mentioned above. Taking these various arguments into account, we consider our first model – whereby pseudoteeth develop after the completion of jaw bone (circumferential) growth – as more plausible than the second one.

### Relationships between pseudoteeth and rhamphotheca, and the phylogenetic affinities of the Odontopterygiformes

Whatever the model supported, the basic structural peculiarities of the pseudoteeth of *Pelagornis mauretanicus* have several functional implications bearing on life history traits and gross ecological adaptations in this species. *Pelagornis* pseudoteeth display an abundant intra-cortical network of vascular canals, and numerous vascular foramina perforating their surface. These features suggest that (1) the periosteum that covered the pseudoteeth and was involved in their growth was highly vascularized, and (2) these capillaries penetrated the cortex *via* the foramina mentioned above. These observations agree with those made by Howard [3] for *P. orri*. Obviously, given the functional constraints related to food grasping by the pseudoteeth, this anatomical organization would have led to an important risk of haemorrhage, if the pseudoteeth were not covered in active life with a tissue mechanically more resistant than the periosteal membrane. In addition, the structural characteristics of the parallel-fibered tissue forming the walls of the pseudoteeth, a kind of bone tissue known to be moderately mineralized [21], [30]–[32] (see also [22]), are indicative of poor rigidity and surface hardness. Such mechanical properties, with further weakening of pseudoteeth by the abundance of intracortical vascular canals, are little compatible with the shear constraints applying to what appears to have functionally replaced a generalized, piscivorous-like dentition [11], [17], used for grasping and holding prey before swallowing it. These features (together with the absence of wear traces that should be expected, e.g., on teeth), suggest that the pseudoteeth, their periosteum, and other associated soft tissues (e.g., mesenchymal tissues) must have been covered in life with a relatively rigid epithelium. This epithelium, in order to help pseudoteeth to sustain functional constraints, needed to be differentiated into a keratinized and hardened rhamphotheca. This conclusion agrees with previous assumptions about *P. orri* [3], [18].

The exact shape of the rhamphotheca and whether it closely paralleled the shape of all the underlying pseudoteeth, or only the more prominent ones, is not known with certainty. It cannot be excluded that the smallest pseudoteeth (ranks 4 and perhaps 3) were embedded in the rhamphotheca and not paralleled by protrusions of the external tomial surface of the latter. However, in any case, the protruding shape of large pseudoteeth was paralleled by the rhamphothecal outline as is attested by the existence of deep fossae on the ventral side of the bony rostrum that accommodated, like furrows, the lower (mandibular) row of pseudoteeth (ranks 1 and 2) at beak occlusion [5], [6], [9]. The larger pseudoteeth with their rhamphothecal covering in life were therefore necessarily as acute as their bony cores are.

Howard [3] proposed that the rhamphotheca was minutely serrated on tomia and pseudoteeth, based on the fluted appearance of some areas of tomia on bony specimens. The argument is not conclusive because a wide number of extant large birds show jaw bone tomia with the same minute anatomical appearance, without showing a serrated rhamphotheca (AL pers. obs.). It nevertheless remains possible that the rhamphothecal tomia was microserrated - which improves grasping efficiency - as is the case in many bird taxa, independently of the shape of underlying bone tomia [2].

A keratinized epithelium, however, could have been an impediment for the growth of the underlying bony pseudoteeth, whatever the growth model considered. Once the rhamphotheca is grown and hardened, it is continuously replaced from the living basal epithelial layer, but keeps a stable general shape. A dramatic shape modification of the hardened rhamphotheca would be required to accommodate the growth of acute underlying bony pseudoteeth. Such cases are unknown in living taxa, and we make the assumption that in pseudotoothed birds the keratinization locally took place subsequent to pseudoteeth growth completion. Among living birds, only a few species show at least locally delayed and/or reduced keratinization of the rhamphotheca. Anseriformes have a semi-rigid rhamphotheca, with the exception of its cranial apex [33]. Members of the Charadriidae (waders, Charadriiformes) and the Apterygidae (kiwis, Apterygiformes) are the only other birds known to have partly or wholly soft rhamphotheca [33], [34]. One phylogenetic hypothesis for the placement of the Odontopterygiformes relative to the other Neornithes places them as the sister group to the Anseriformes, altogether constituting the Odontoanserae [12]. The other current hypotheses make them either sister to Galloanserae (which comprises Anseriformes and Galliformes) or perhaps suggest by default a branching at the base of Neognathae [14]. Most recent cladistic analyses seem to favor a position as stem Galloanserae, or even stem Neognathae, but acknowledge an important uncertainty [14]. In addition, several characters are shared with *Hesperornis* and *Ichthyornis*, and some with stem Palaeognathae; these characters being interpreted as plesiomorphic or convergent. Our inferences regarding the rhamphotheca, combined with evidence from extant birds, tend to support the hypothesis of a close phylogenetic relationship between Odontopterygiformes and Anseriformes. Interestingly, independent evidence from paleoneurology also provides some characters supporting this phylogenetic relationship (long anastomosis intercarotica and enclosure of the carotid rami), while other characters are ambiguous [13]. In this respect, a partly soft rhamphotheca, at least locally until late stages of somatic growth, might even prove to be a synapomorphy of these two clades. It is also significant that the only other known birds that ever had bony odontoids are the recently extinct Hawaiian moa-nalos [16], which were insular endemic Anseriformes derived from a duck species of the tribe Anatini [35].

### Inference for altriciality

According to our interpretation, the full keratinization and hardening of the epithelium must have occurred subsequent to the completion of pseudotooth growth, at least in the precise jaw spots where the pseudoteeth developed. Given the shearing constraints that would have been associated with functionality, it is unlikely that immature pseudoteeth with soft covering epithelium were able to cope with the requirements of efficient foraging activity for a piscivorous bird (*sensu lato*, i.e., diet composed of small marine animals). They would therefore have become functional after full growth of pseudoteeth bony cores (whatever the timing of this growth) and overlying epithelium keratinization, a situation that would have occurred well after hatching, given the large overall body size and jaw size in *Pelagornis*, and eggshell size limitation. In addition, in our preferred hypothesis of pseudoteeth growth, the latter would have been completed with even more delay. Therefore, the functionality of the feeding apparatus must have been much delayed, which implies in turn a prolonged dependence on parental care, notably for food supply. If so, *Pelagornis mauretanicus* would have been a highly altricial species, with a long period of inability of the chicks to feed by themselves. For instance, the parents might have fed chicks by regurgitation during an extended period of time. This would have conveniently provided the soft food needed for the chicks. Incidentally, there is no direct relationship inferred between late keratinization of rhamphotheca *per se* and altriciality. The particular factor suggesting enhanced altriciality in pseudotoothed birds is that late keratinization was associated with the acute shape and fragile constitution of pseudoteeth. Hence, in juveniles the pseudodentition (bone and rhamphotheca considered altogether) would have been too fragile to support shear strain or stress during food acquisition. In addition, during feeding activity, high local strain on the acute peaks of bony pseudoteeth through a non-sufficiently hardened covering epithelium might have resulted in epithelial lesions.

An alternative hypothesis would be that the diet of the young might have been different from that of adults, and softer, thus allowing them to feed by themselves. However, this hypothesis raises such a broad series of unanswered questions on ecology, morphofunctional adaptations in young individuals, etc., that our hypothesis of altriciality seems at present more convincing, although it is not yet verifiable. Future independent analyses (e.g., isotopes for diet; *in silico* analyses of resistance of jaws to strain and stress, etc.) should shed some light on this question.

The phylogenetic placement of Odontopterygiformes as sister taxon to Anseriformes or to Galloanserae would support the hypothesis that they evolved from precocial ancestors, since palaeognaths, waterfowl and landfowl are precocial [36]. Therefore, pseudotoothed birds likely developed altriciality from precocial ancestors, convergently with birds like albatrosses and other Procellariiformes, the closest relatives of the latter being also precocial [36], according to some phylogenies [37]. Pseudotoothed birds also converged upon albatrosses in other aspects, such as wing design [11].

## Conclusions

Our histological and micro-anatomical analyses show that the pseudoteeth of *Pelagornis mauretanicus* are bony excrescences in the form of an empty pseudo-cone, or a blade (depending on pseudotooth rank), on the occlusal surface of the jaw bone cortex. Histological details suggest two alternative hypotheses to explain pseudotooth growth. The most convincing one is that the pseudoteeth in *Pelagornis mauretanicus* developed relatively late (final stages of somatic growth) as the consequence of a simple, local protraction in the osteogenic activity of periosteal osteoblasts. This process occurred when the growth in diameter or thickness of jaw bones was completed. The absence of any dental tissue is confirmed, as well as the hypothesis that pseudoteeth were covered in life by the rhamphotheca. The latter could only become hardened after completion of pseudoteeth growth locally. Presumably, the young could use their beak for foraging and feed independently only once the rhamphotheca was mature, which means late after hatching, for all these reasons. Hence, *P. mauretanicus* (as well as probably other pseudotoothed birds) was likely a very altricial species, a derived condition considering the more likely phylogenetic positions of Odontopterygiformes, rather basal among the Neognathae or among the Galloanserae. Among these hypotheses, a sister-relationship with Anseriformes finds further support from our results, because the latter clade is among the few that comprise species with soft rhamphotheca, or delayed hardening of the rhamphotheca, at least locally, as we infer for *P. mauretanicus*.

## Supporting Information

Table S1 **Quantitative values of the specimens shape and structure.** The measurements are indicated for individual pseudoteeth and specimens. All in mm unless stated otherwise. PT, pseudotooth. c-c, cranio-caudal. l-m, latero-medial. ^a^from occlusal edge of basal plate to tip. ^b^estimated total height. ^c^compactness measured on virtual slices of the rank 3 PT of AaO-PT-A, as: [(bone section surface S minus vascular canal areas)/bone section surface S] ×100.
(DOCX)
