# Supplementary material for: Structure and Growth Pattern of Pseudoteeth in Pelagornis mauretanicus (Aves, Odontopterygiformes, Pelagornithidae)
Source: PLoS One. 2013 Nov 14;8(11):e80372. doi: 10.1371/journal.pone.0080372 (PMC3828250; doi:10.1371/journal.pone.0080372)
Supplement: Table S1 — Quantitative values of the specimens shape and structure. The measurements are indicated for individual pseudoteeth and specimens. All in mm unless stated otherwise. PT, pseudotooth. c-c, cranio-caudal. l-m, latero-medial. afrom occlusal edge of basal plate to tip. bestimated total height. ccompactness measured on virtual slices of the rank 3 PT of AaO-PT-A, as: [(bone section surface S minus vascular canal areas)/bone section surface S] ×100. (DOCX) [file pone.0080372.s001.docx]

| **Measure** | **PT3 of AaO-PT-A** | **2 PTs4 of AaO-PT-A** | **PT2 of AaO-PT-B** | **PT4 of AaO-PT-B** | **PT1 of AaO-PT-C** |
| --- | --- | --- | --- | --- | --- |
| PT Height^a^ | 7.9 (9.2e)^b^ | 3.5 (?)^b^; 5.4 (6.7e)^b^ | 10.7 (12.5e)^b^ | 2.1 (3.9e)^b^ | 16.6 (19.8e)^b^ |
| Max. cranio-caudal diameter of PT base | 3.6 | 1.1-1.7; 1.7 | 5.0 | ca. 1.8 | ca. 9.1 |
| Max. latero-medial diameter of PT base | ? | ?; 2.7 | 6.5 | 3.3 | 8.1 |
| Ratio cranio-caudal/latero-medial diameters of PT base | ? | 0.63 | 0.77 | 0.55 | 1.12 |
| Cortex thickness of pseudotooth near apex | 0.2-0.45 | 0.15-0.25 (c-c) | 0.25-0.7 | 0.2 | 0.4-0.9 |
| Cortex thickness of pseudotooth near base | 0.3-0.4 | 0.2-0.45 (c-c) | 0.45-0.7 | 0.5-0.65 | 0.3-0.8 |
| Cortex thickness of basal plate | 0.7-0.75 | x | 0.55(middle)-1.4(border) | x | 0.25-0.9 |
| Cortex thickness of adjacent other main jaw bone | 0.5-0.85 | | 0.55-1.35 | | 0.65-0.85 |
| Diameter of basal foramen | ? | x | 0.35(l-m) x 0.55(c-c) | x | 1.2(l-m) x 2.2(c-c) |
| Cortical compacities^c^ of : PT apex; PT base | 89.3 %; 90.4 % |  | | | |
| Cortical compacities^c^ of: basal plate; adjacent jaw bone | 96.8 %; 95.1 % |  |  |  |  |
